# Challenges with achieving and maintaining oral cholera vaccine coverage: insights from serial cross-sectional representative surveys in a cholera-endemic community in the Democratic Republic of the Congo

**DOI:** 10.1136/bmjph-2024-001035

**Published:** 2025-01-19

**Authors:** Aybüke Koyuncu, Patrick Musole Bugeme, Juan Dent, Chloe Hutchins, Hanmeng Xu, Karin Gallandat, Oliver Cumming, Joseph Matundanya, Aimé Cikomola, Delphin Rukakiza, Merveille Nkombo, Jaime Saidi Mufitini, Baron Bashige Rumedeka, Laurent Akilimali, Elizabeth C Lee, Placide Okitayemba Welo, Jackie Knee, Andrew S Azman, Espoir Bwenge Malembaka

**Affiliations:** 1 Department of Epidemiology, Johns Hopkins Bloomberg School of Public Health, Baltimore, Maryland, USA; 2 Center for Tropical Diseases and Global Health (CTDGH), Université Catholique de Bukavu, Bukavu, Congo (the Democratic Republic of the); 3 Department of Disease Control, London School of Hygiene and Tropical Medicine, London, UK; 4 Programme Elargi de Vaccination, Ministère de la Santé Publique, Hygiène et Prévention, Bukavu, Congo (the Democratic Republic of the); 5 World Health Organization, Kinshasa, Congo (the Democratic Republic of the); 6 Zone de Santé d'Uvira, Ministère de la Santé Publique, Hygiène et Prévention, Uvira, South Kivu, Congo (the Democratic Republic of the); 7 Programme National d’Elimination du Choléra et de Lutte Contre les autres Maladies Diarrhéiques (PNECHOL-MD), Ministère de la Santé Publique, Hygiène et Prévention, Kinshasa, Congo (the Democratic Republic of the); 8 Geneva Centre for Emerging Viral Diseases and Division of Tropical and Humanitarian Medicine, Geneva University Hospitals, Geneva, Switzerland

**Keywords:** Vaccination, Disease Outbreaks, Primary Prevention, Disease Transmission, Infectious, Preventive Medicine

## Abstract

**Background:**

We conducted three serial cross-sectional representative surveys after a mass cholera vaccination campaign in Uvira, Democratic Republic of the Congo to (1) estimate the vaccination coverage and explore heterogeneity by geographic and demographic factors; (2) examine barriers and facilitators of vaccine uptake and (3) describe the changes in coverage over time and predict future coverage.

**Methods:**

We collected data on sociodemographics, self-reported vaccination status, population movement and knowledge, attitudes and behaviours related to killed oral cholera vaccines (kOCVs) in August 2021, April 2022 and April 2023, approximately 11, 19 and 30 months postvaccination. We compared the characteristics of participants by vaccination status and explored the potential role of population movement as a cause for low coverage. We used an exponential decay model to predict the proportion of the population vaccinated with ≥1 dose of kOCV over time based on age-specific coverage.

**Results:**

We enrolled 8735 participants from 1433 households across all surveys. Coverage in survey 1 (August 2021) was 55% for ≥1 dose of kOCV (95% CI 51 to 60) and 23% for ≥2 doses (95% CI 20 to 27). Vaccine refusal was associated with a lack of confidence in the vaccine’s safety, and 29% of unvaccinated adults reported it was unlikely they would accept kOCVs if an additional mass vaccination campaign was conducted in their area. Coverage of ≥1 one dose of kOCV declined on average by 18% per year (95% credible interval 14 to 23) and was 39% (95% CI 36 to 43) by survey 3 (approx. 30 months after second dose campaign).

**Conclusions:**

Our findings suggest that in settings like Uvira, efforts to strengthen vaccine confidence are needed to achieve higher campaign coverage, and vaccine coverage dilution may be reduced by more frequent and coordinated geographic vaccination efforts.

**WHAT IS ALREADY KNOWN ON THIS TOPIC**

- Killed oral cholera vaccines (kOCVs) are an effective tool for preventing and controlling the spread of cholera. Achieving and maintaining high vaccine coverage is key to protecting communities through both direct and indirect protection.

**WHAT THIS STUDY ADDS**

- Coverage of at least 1 dose of kOCV was lower than in pre-COVID-19 cholera vaccination campaigns conducted in the Democratic Republic of the Congo and elsewhere and declined on average by 18% per year (95% credible interval: 14–23).
- Investing in strategies that strengthen vaccine confidence is critical for improving the uptake of kOCVs in epidemic-prone settings like Uvira, given that 30% of unvaccinated individuals reported being unsure or little or not at all likely to accept kOCVs if an additional campaign was conducted.

**HOW THIS STUDY MIGHT AFFECT RESEARCH, PRACTICE OR POLICY**

- Substantially interrupting cholera transmission in urban cholera hotspots, like Uvira, may require more frequent preventative campaigns and strategies that improve the uptake of kOCVs in future campaigns.
- Synchronous vaccination campaigns, sometimes across national borders (e.g., in Burundi in the case of Uvira), may be beneficial to prolonging the indirect protection from kOCVs, ideally leading to revaccination campaigns occurring on a similar timescale to the waning of direct protection (i.e., 5 years or more).

## Background

 While improvements to water and sanitation infrastructure are needed for long-term cholera control and elimination, killed oral cholera vaccines (kOCVs) are an effective short-term tool for prevention and control.^1^ kOCVs are typically administered as a two-dose regimen at least 14 days apart and confer protection against cholera for at least 5 years.^2^ In 2013, the World Health Organization (WHO) and partners established a global stockpile of kOCVs to facilitate rapid access to them in emergency outbreak response, which was later expanded to both emergency and preventative use.^1^ In 2022, 72 million doses of kOCV were requested from the global stockpile for preventative and reactive vaccination campaigns, and 33 million doses were delivered to requesting countries.^3^ In response to increased demand for kOCVs in 2022, the International Coordinating Group managing the emergency stockpile recommended changing the standard two-dose vaccination regimen to a single-dose strategy in emergency campaigns.^4^ Maximising the benefits of the limited supply of vaccines requires understanding the barriers to achieving high population coverage during vaccination campaigns as well as factors that may influence coverage over time.

The goal of preventive vaccination campaigns is to reduce the incidence of cholera and the occurrence of outbreaks. This is likely achieved through both direct protection conferred by the vaccines and indirect protection of unvaccinated through herd immunity.^5 6^ While kOCVs are relatively easy to deliver, given that they are oral and fairly heat stable, achieving high two-dose coverage can be challenging, especially in low-resource settings where cholera tends to occur, due to the short and often hastily planned vaccination windows and vaccine-related misinformation, particularly in the post-COVID-19 era. Even when high coverage is achieved, high rates of migration in and out of urban and periurban areas combined with the highly focal nature of kOCV campaigns can lead to rapid decay in the effective vaccine coverage in the population.^7^ Understanding the drivers of initial vaccine coverage and subsequent decay can help inform locally tailored vaccination and revaccination plans in cholera endemic settings.

In 2020, the Democratic Republic of the Congo (DRC)’s Ministry of Health organised two preventative mass cholera vaccination campaigns with Euvichol-Plus in five health zones of South Kivu province, including Uvira. The target population for the campaigns was all persons ≥1 year of age, residing in the Uvira Health Zone. The campaign was initially planned as a preventive campaign but was implemented with an accelerated timeline as an emergency campaign in response to flooding. Door-to-door mobile vaccination teams and fixed points were set up from 29 July to 8 August 2020 (first round) and from 28 September to 5 October 2020 (second round). Understanding key successes and challenges of achieving and maintaining high kOCV coverage using mass vaccination campaigns in urban settings such as Uvira can provide critical information to help optimise future campaigns and prevent and control cholera outbreaks.

Here, we used data from three serial cross-sectional representative surveys conducted 10–30 months after vaccination to (1) estimate the postvaccination coverage and explore heterogeneity by geographic and demographic factors; (2) examine barriers and facilitators of vaccine uptake and (3) describe the changes in kOCV coverage over time and predict the likely trajectory of future coverage.

## Methods

### Setting

The city of Uvira (designated as Uvira throughout this document) is an urban area of approximately 300 000 inhabitants, located in the broader Uvira Health Zone, on the northwestern shore of Lake Tanganyika. Essentially a trading city, Uvira is home to the port of Kalundu, the second largest port in the DRC, and serves as a regional hub for trade between the country and Burundi, Tanzania, Zambia, and with DRC towns of Kalemie, Baraka and Moba which are known to be major fishing and mining sites. Cholera has been endemic in Uvira since the 1970s when the first ever documented use of a cholera vaccine occurred in response to a large outbreak in eastern DRC.^8^ Uvira health zone has been classified as a cholera transmission hotspot by both the DRC national cholera programme and international agencies and is targeted for reactive and preventive vaccination campaigns.^9^,^11^

### Sampling

As part of a study on the impact of mass cholera vaccination, we conducted three population-representative household surveys in Uvira. Survey round 1 was conducted in August 2021 (approx. 11 months after the second round of the vaccination campaign), round 2 occurred in April–May (approx. 19 months after the campaign) and round 3 occurred in April–May 2023 (approx. 30 months after the campaign). To create a sampling frame, we identified all structures built within the boundaries of Uvira using high-resolution satellite imagery captured between 26 February and 16 March 2020, by Pleiades P1A (Airbus Intelligence, Toulouse, France). Through an iterative process of machine learning and manual verification of the imagery, 59 065 structures were identified as potential dwellings.^12^ We excluded 495 structures with surface areas >500 m^2^, on the assumption that they are unlikely to be residential structures. From the remaining 58 570 structures, we randomly sampled structures to visit for each of the three surveys. In the second round of survey, we removed from the sampling frame an additional 135 structures identified as non-residential in the first round of survey, and in round 3, we removed a further 172 non-residential structures identified in the second round of survey to improve our sampling efficiency.

Our household surveys were originally designed to allow accurate estimation of vaccination coverage in Uvira as well as cholera seroincidence. We estimated that 2120 participants from 530 households would be sufficient to achieve statistical power of 90% to estimate the coverage of ≥1 kOCV dose of 70%±4.6%, assuming a household design effect of 2 (intracluster correlation coefficient at household level=0.4). Survey round 1 focused on vaccination coverage alone, while the following surveys embedded a serological data collection (serological data not presented here). In those serosurveys, we enrolled all consenting individuals in households where at least one member accepted to provide a blood sample. That led to a far greater number of individuals and households enrolled than the calculated sample size.

### Data collection

Study teams were provided a list of geographic coordinates (presumed households) to visit each day and approached the closest residential door within 20 m of the point, which could have been in any direction from the sampled geographic coordinate. Teams used the mobile app OsmAnd to locate each household. If no one was available (typically the head of household or delegate), study staff made up to three additional attempts to visit the household to offer enrolment in the study. We drew replacement points for all cases where the originally sampled coordinate corresponded to a non-residential structure, was no longer there, or was no longer occupied. Replacement points were only used at the end of the survey if the target sample size was not reached.

Upon visiting a randomly selected household, all consenting individuals aged at least 1 year were eligible to participate. Though the study is not designed to follow individuals over time (as these are serial cross-sectional surveys), participation in a prior survey did not disqualify an individual from participating in a future round if selected by chance.

Trained surveyors administered pretested electronic questionnaires, available in French and Kiswahili, to consenting participants using the Open Data Kit. Participating household heads completed a household questionnaire that collected data on household composition over time, infrastructure (including water, sanitation and hygiene (WASH) and assets) and births and deaths. Individual questionnaires with each household member were used to collect data on demographics, how long households have lived in their current residence and where they last lived (round 1 only), history of kOCV vaccination, routine vaccination of children under 2 years of age (round 1 only) and behaviours related to care seeking and WASH.

Cholera vaccination status was ascertained using a series of structured questions designed to understand the number of doses an individual received. Before asking each participant whether they were vaccinated, surveyors used visual aids and described the vaccine presentation, mode and timing of administration to help distinguish it from other vaccines. Participants reporting to have been vaccinated were asked for dose and delivery details, as well as their vaccination card for visual verification.

In the second round of the survey, we added a series of questions to the individual questionnaire from a validated tool to better understand barriers and enablers of vaccination and to examine individual knowledge, attitudes and behaviours related to kOCV.^13^ We collected data on perceptions about cholera disease risk and severity, confidence in vaccine safety and effectiveness, perceptions about kOCV uptake among family, friends and community members, types of trusted information sources and future vaccination intentions (likelihood of accepting kOCVs if another campaign was conducted in the future). Questions about vaccine sentiments were only asked to adults aged 18 or older.

### Analysis

Our primary outcome was the proportion of the total population that reported receiving at least one dose of kOCV (henceforth, coverage). Analyses of coverage by sociodemographic variables (e.g., age, sex) were restricted to round 1 of the survey to minimise the influences of demographic changes and population movement on conclusions about campaign performance. For coverage estimates, the clustering of individuals in the same household was accounted for by including weights for the household size with the use of the ‘survey' package in R. Vaccination coverage in the population at any point in time is both a function of the performance of the initial vaccination campaign and changes in coverage due to births, deaths and population movement. Immigration of vaccinated individuals and/or emigration of unvaccinated individuals from Uvira can both contribute to coverage decline over time. As round 1 was conducted approximately 11 months after the vaccination campaign ended, we also aimed to estimate the range of plausible coverage estimates just after the vaccination campaign. We used an exponential decay model to predict the proportion of the population vaccinated with at least one dose of kOCV over time by age:


$$v\left(t,g\right)\;=\;v_{0g}\;\times \;e^{-\lambda_{g}t}$$

Where v(t,g) is the coverage at time t for age group g, v_0g_ is the initial campaign coverage for age group g and $\lambda_{g}$ is the age-specific monthly decay rate. Data from each survey were assumed to follow a binomial distribution, and this uncertainty was propagated into our estimates of the decay rate and coverage over time through a model coded in Stan.^14^ We estimated the overall population coverage at time t by calculating a weighted average of age-specific coverage at each time point based on the age distribution in the full study population from all survey rounds.

As decreases in population coverage of the vaccine are caused by several demographic processes, including births, deaths and population movement, to better understand the drivers of decline, we conducted descriptive analyses of questions from the household survey on population movement into and out of Uvira (online supplemental material S2). Data on population movement included all movement within and outside of Uvira in round 1 but included only movements out of or into Uvira from areas outside of the town in rounds 2 and 3.

To help visualise coverage across the city, we created smoothed coverage maps using binomial generalized additive models implemented with the *mgcv* R package to estimate the predicted probability of receiving at least one dose of kOCV in Uvira. We used the mean of the predicted probability of 0.1 km by 0.1 km grid cells within each neighbourhood to estimate the neighbourhood-level coverage of at least one dose of kOCV.

To understand barriers and facilitators of kOCV uptake at the individual level, we calculated the proportion of age-eligible individuals who self-reported receiving ≥1 dose of kOCV stratified by age, sex, education level, wealth quintile and residence health area. We compared vaccine sentiments and future vaccination intentions between individuals who received ≥1 dose and individuals who were unvaccinated using χ^2^ tests. We estimated household socioeconomic status by creating a composite wealth index using principal component analysis of household assets (e.g., ownership of cell phone, refrigerator, etc.^15^) and housing characteristics (e.g., building material of walls and floors).^16^ The household wealth index was attributed to each household member, then divided in quintiles. In sensitivity analyses, we compared vaccine sentiments between individuals who received only one dose to those who received two doses.

We used R version 4.3.0 for all analyses and followed the Strengthening the Reporting of Observational studies in Epidemiology guidelines for presenting results (online supplemental material S1). Data and code needed to reproduce the primary analyses in this paper are available at https://osf.io/2quwy/.^17^

### Ethics

Written informed consent was obtained from all study participants. Child assent was also obtained for minor participants (aged 7–17 years), after written consent from a parent or adult guardian. The study was approved by the ethics committee at the Johns Hopkins Bloomberg School of Public Health (IRB00015785), the London School of Hygiene and Tropical Medicine (25365) and the University of Kinshasa School of Public Health (ESP/CE/65/2021).

### Patient and public involvement

Patients or the public were not involved in the design, conduct, reporting and dissemination of this study.

## Results

We enrolled 2292 individuals in round 1 (383 households), 3579 individuals in round 2 (622 households) and 2864 individuals in round 3 (429 households). A majority of participants (62%) were <20 years of age at the time of the survey and all survey rounds had a higher proportion of females compared with males (55% female overall) (table 1). Based on the reported age at the time of the survey and the date of each survey round, we estimate 16% of participants (n=1364) were 1–4 years of age and 7% of participants (n=585) were <1 year of age at the time of the vaccination campaign. Our sample in each survey round had a slightly higher proportion of females compared with the 2021 official population data in Uvira (50%), but a similar proportion of participants <5 years of age and 50 years of age and older.^18^ The median household size was eight individuals and was stable across survey rounds (table 1).

**Table 1:** Sociodemographic characteristics of study participants and household characteristics by survey round, Uvira, 2021–2023

Individual characteristic (col %)	Overall (n=8735)	Round 1 (n=2292)	Round 2 (n=3579)	Round 3 (n=2864)
Sex*				
Female	4761 (55)	1231 (54)	1942 (54)	1588 (55)
Male	3970 (45)	1057 (46)	1637 (46)	1276 (45)
Age at time of survey†				
1–4	1246 (14)	318 (14)	513 (14)	415 (14)
5–9	1607 (18)	406 (18)	656 (18)	545 (19)
10–19	2624 (30)	674 (30)	1048 (30)	902 (32)
20–34	1696 (19)	455 (20)	706 (20)	535 (19)
35–49	846 (10)	234 (10)	351 (10)	261 (9)
50+	704 (8)	198 (9)	301 (8)	205 (7)
Highest level of education‡				
None or less than primary	2946 (39)	609 (31)	920 (30)	1417 (58)
Primary	2183 (29)	737 (38)	1161 (38)	285 (12)
Secondary	1982 (26)	529 (27)	827 (27)	626 (26)
Bachelors	360 (5)	90 (5)	152 (5)	118 (5)
Other	9 (0.1)	0 (0)	9 (0.3)	0 (0)

Household characteristic N (col %)	Overall (n=1434)	Round 1 (n=383)	Round 2 (n=622)	Round 3 (n=429)
Household size, median (IQR)	8 (6–11)	7 (6–10)	8 (6–11)	8 (6–11)
Number of people per bedroom, median (IQR)	3 (2–5)	3 (2–5)	3 (2–5)	3 (2–5)
Wealth quintile				
First (lowest)	346 (24)	72 (18)	140 (23)	136 (32)
Second	375 (26)	105 (27)	160 (26)	111 (26)
Third	409 (29)	112 (29)	186 (30)	111 (26)
Fourth	211 (15)	63 (16)	94 (15)	55 (13)
Fifth (highest)	89 (6)	31 (8)	42 (7)	16 (4)
Drinking water source§**				
Improved	979 (69)	260 (71)	317 (51)	309 (72)
Unimproved	437 (31)	108 (29)	303 (49)	120 (28)
Type of toilet¶**				
Improved	1016 (72)	249 (67)	383 (58)	331(78)
Unimproved	397 (28)	121 (33)	283 (42)	94 (22)

Drinking water sources and type of toilet were classified as improved or unimproved based on the criteria from the Joint Monitoring Programme for Water Supply, Sanitation and Hygiene (JMP^29^).

In rounds 2 and 3, individuals who did not consent to provide a blood sample were asked a shorter version of the questionnaire, which only collected data on sociodemographic characteristics and vaccination status.

* Missing for n=4 participants in round 1

† Missing for N=7 participants in round 1, N=4 participants in round 2 and N=1 participant in round 3.

‡ Missing for N=327 participants in round 1, N=510 participants in round 2 and N=418 participants in round 3.

§ Missing for N=15 households in round 1 and N=3 households in round 2

¶ Missing for N=13 households in round 1, N=4 households in round 2 and N=4 households in round 3.

** Drinking water source and type of toilet were assessed at the household-level for survey rounds 1 and 3 but at the individual-level for round 2. For round 2, households were categorised as having unimproved drinking water/toilets if any person in their household reported an unimproved water/toilet.

### Population coverage

Vaccine coverage in round 1 was 55% for ≥1 dose of kOCV (95% CI 51 to 60; design effect: 4.6) and 23% for ≥2 doses (95% CI 20 to 27; design effect: 4.3). At the time of the survey, 93% of participants had been eligible for vaccination at the time of the campaign (aged 1 year or older in 2020), and population-level coverage and age-eligible population-level coverage were similar (55% vs 56% for at least one dose, 23% vs 24% for at least two doses).

**Figure 1:**
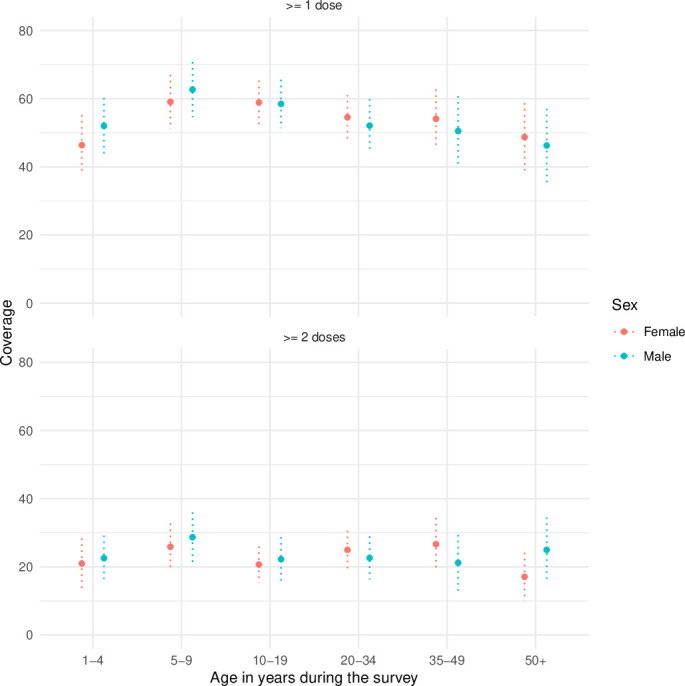
Coverage of oral cholera vaccine by age during the survey and sex in round 1 (10 months after the campaign), Uvira, 2021.

Coverage with at least one dose was similar between females and males (figure 1). Coverage was lowest for adults aged 50 years or older (48%; 95% CI 40 to 56; design effect: 1.3) and highest in children 5–9 years (61%; 95% CI 54 to 67; design effect: 1.9).

Coverage was inversely associated with wealth index (χ^2^ p<0.001), with the lowest coverage in the wealthiest quintile (50%; 95% CI 35 to 64) and highest in the least wealthy quintile (63%; 95% CI 53 to 74). We found large disparities in coverage between neighbourhoods. In round 1, coverage of at least one dose ranged from 23% (95% CI 10 to 36) in Nyamianda, the wealthiest neighbourhood home to the city council, to 78% (95% CI 50 to 107) in Kibondwe where the 2020 flooding and frequent rise in the level Lake Tanganyika have caused notable damage to housing and WASH infrastructures. Only 22% of individuals who reported receiving at least one dose of kOCV had vaccination cards available at the time of the interview, and most participants had lost their cards prior to the interview (75%).

### Barriers and facilitators of vaccine uptake

Vaccine refusal was associated with a lack of confidence in vaccine safety and perceived importance of cholera vaccine in providing protection against cholera, but was not strongly related to perceptions about disease severity or risk of getting infected with cholera (table 2). There were no differences in the uptake of at least one dose of kOCV (p=0.37), perceptions about the likelihood of getting sick with cholera (p=0.21) or perceptions about the severity of cholera (p=0.09) based on individual history of diarrhoea in the last month.

**Table 2:** Perceptions about cholera and cholera vaccines between unvaccinated (0 doses) and vaccinated (at least one dose) adults, Uvira, 2022

% (N)	Unvaccinated	Vaccinated	χ^2^ p value
I will probably get sick with cholera			
Strongly agree	82% (578)	86% (461)	0.13
Somewhat agree	15% (102)	11% (61)	
Neither agree nor disagree	3% (22)	2% (11)	
Don't agree	0% (0)	0% (0)	
Getting sick with cholera can be serious			
Strongly agree	81% (650)	90% (581)	<0.001
Somewhat agree	17% (136)	9% (55)	
Neither agree nor disagree	3% (22)	2% (11)	
Don't agree	0% (0)	0% (0)	
How important is a cholera vaccine to protect you against cholera?			
Very important	69% (579)	91% (615)	<0.001
Moderately important	19% (156)	7% (50)	
Little important	6% (53)	1% (7)	
Not important	6% (50)	0% (2)	
How safe do you think a cholera vaccine is for you?			
Very safe	56% (390)	74% (456)	<0.001
Moderately safe	31% (219)	21% (128)	
Little safe	12% (87)	5% (30)	
Not at all safe	0% (0)	0% (0)	
How concerned are you that a cholera vaccine could cause you to have a serious* reaction?			
Not concerned	39% (309)	65% (424)	<0.001
Little concerned	14% (116)	8% (55)	
Moderately concerned	24% (194)	16% (105)	
Very concerned	23% (183)	22% (72)	
How much do you trust the public health agencies that recommend the cholera vaccine?†			
Fully trust	54% (199)	74% (246)	<0.001
Mostly trust	31% (115)	21% (70)	
Somewhat trust	10% (38)	5% (17)	
Do not trust	5% (19)	0% (1)	
Perceptions about how many family members are vaccinated‡			
Almost all	5% (28)	25% (160)	<0.001
Many	15% (94)	40% (255)	
Somewhat agree	46% (280)	34% (216)	
None	34% (211)	1% (8)	
Perceptions about how many community and religious leaders are vaccinated§			
Almost all	3% (10)	11% (47)	<0.001
Many	10% (36)	30% (126)	
Somewhat agree	56% (201)	48% (201)	
None	31% (109)	11% (46)	

* Serious means you would not be able to perform your daily activities

† Only asked to subset of participants who were familiar with public health agencies recommending vaccines (n=373 unvaccinated and n=334 vaccinated).

‡ The question was: ‘If you had to guess, about how many of your family and friends have received a cholera vaccine?’

§ The question was: ‘If you had to guess, about how many of your community leaders or religious leaders have received a cholera vaccine?’

Adults who received only one dose of cholera vaccine had mostly similar perceptions about cholera and cholera vaccines, though a higher proportion of adults who received at least two doses of cholera vaccine believed almost all of their family members (p=0.002) and community and religious leaders (p=0.04) were vaccinated compared with adults who only received one dose (online supplemental material S3).

Among individuals who reported not receiving any doses of cholera vaccine, the top three reasons for non-vaccination were suspecting the vaccine contained COVID-19, Ebola or other microbes (35%), being away from home when vaccinators came (26%), and fear of side effects (18%) (online supplemental material S4). A higher proportion of vaccinated individuals reported having seen or heard rumours about cholera vaccines (86% vs 68%; p<0.001). Among individuals who reported seeing or hearing rumours (76%; n=1171 of 1545), rumours were most frequently heard/seen from family members (53%), other household members (48%), in community meetings (40%), on the radio (22%) and/or on social media (19%). Primary care providers were the most trusted sources of information about cholera vaccines and were a trusted source for both unvaccinated adults (57%) and vaccinated adults (64%).

If an additional mass cholera vaccination campaign was conducted in their area, 29% of unvaccinated adults reported it was unlikely (little likely or not at all likely) they would accept cholera vaccines while 71% reported it was very or moderately likely. Individuals whose reasons for being currently unvaccinated were related to vaccine access (e.g., being away from home at the time of the campaign) were more likely to accept the vaccine if an additional campaign was conducted compared with individuals who reported reasons for non-vaccination related to vaccine confidence (e.g., fear that the vaccine was containing the COVID-19, Ebola or other microbes).

In contrast, only 9% of adults who were vaccinated with at least one dose of kOCV at the time of the survey reported it was unlikely they would take cholera vaccines in a future campaign (χ^2^ p value <0.001). Among individuals who were unvaccinated, there was heterogeneity in intentions to get vaccinated in a future campaign by neighbourhood.

### Changes in coverage over time

**Figure 2:**
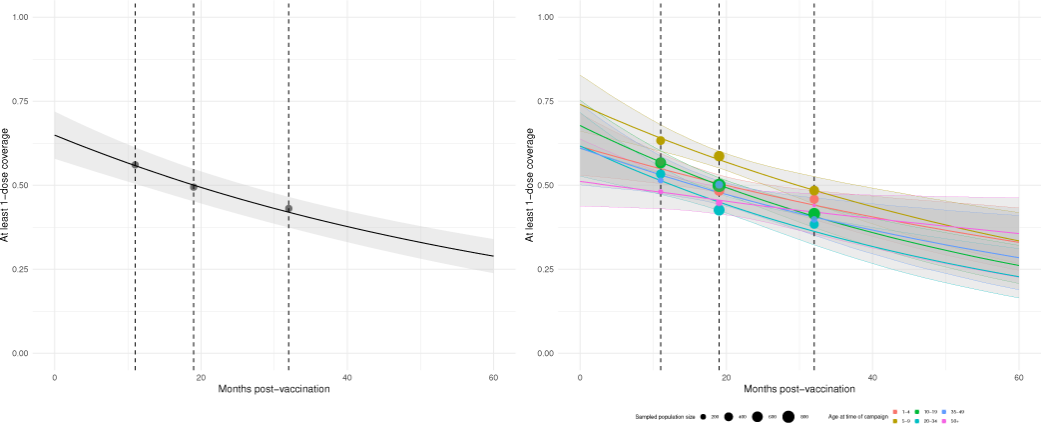
Projected coverage of at least one dose of killed oral cholera vaccine over time for the overall population and by age, Uvira, 2021–2023. Points indicate overall and age-stratified survey coverage estimates with size representing the sample size in each group. Dashed lines indicate survey rounds.

Coverage of ≥1 dose declined to 47% (95% CI 44 to 50) in the second survey round and 39% (95% CI 36 to 43) in the third survey round (table 3). Coverage of exactly one dose declined to 27% (95% CI 24 to 29) in the second survey round and was 28% (95% CI 25 to 31) in the third survey round (online supplemental material S5). Less than 3 years after the vaccination campaign, at the time of the third survey round, only 10% (95% CI 8 to 12) of the population was fully vaccinated against cholera. Based on these data, we estimate that the initial campaign coverage with at least one dose was 66% (95% credible interval: 59 to 74) and coverage with at least two doses was 41% (95% credible interval: 32 to 50) (figure 2).

**Table 3:** Killed oral cholera vaccine coverage from all three surveys, Uvira, 2021–2023

Round	N	Months after second dose campaign[Table-fn T3_FN1]	At least one dose (95% CI)	At least two doses (95% CI)
Survey 1	2292	10.1–10.7	55% (51–60)	23% (20–27)
Survey 2	3583	17.9–18.9	47% (44–50)	20% (18–23)
Survey 3	2864	29.8–30.5	39% (36–43)	10% (8–12)

* The second dose campaign was completed on 05 October 2020.

The proportion of neighbourhoods in Uvira with <50% coverage of at least one dose increased from 36% (5 of 14 neighbourhoods) in round 1 to 71% in rounds 2 and 3 (onlinesupplemental materials S6  S7). Predicted coverage did not consistently decline over time in each grid cell, and coverage in some grid cells increased over time, which is likely an artifact of sampling. The median grid-cell-level coverage in rounds 2 and 3 were 13% (IQR: −10%–30%) and 32% (IQR: 16%–57%) lower than the median coverage in round 1. As with the grid-cell-level analyses, coverage did not consistently decline over time by neighbourhood, and a large decline in coverage between survey rounds 1 and 2 was not strongly predictive of a large decline between rounds 2 and 3 (correlation coefficient=−0.5).

Coverage of at least one dose of kOCV declined on average by 18% per year (95% credible interval: 14 to 23). Assuming a constant rate of coverage loss in the population and no additional vaccination activities, we expect that only 27% of the population will have been vaccinated with ≥1 dose (95% credible interval: 22 to 32) (figure 2) and 4% of the population will have been vaccinated with ≥2 doses (95% credible interval: 1 to 8) (online supplemental material S8) 5 years after the initial campaign (October 2020). The yearly rate of decline for one or more doses was lowest among adults 50 years or older at the time of the campaign (9%; 95% credible interval: 0.7 to 22), potentially reflecting their lower mobility rates.

Based on survey questionnaires, the household net migration rate in the last year was relatively stable across survey rounds and ranged between −12.4 (round 1) and −13.7 (round 3) per 1000 individuals, suggesting more people who were or had been part of surveyed households left Uvira during this period compared with those who migrated into the city. Among households with available data on when they moved to their current home/compound and where they moved from in round 1 (n=275 of 382 households), at the time of the first vaccination campaign (July 2020) 81% of households were living in Uvira health zone and 94% were living in South Kivu province.

## Discussion

Coverage of kOCV was lower than in previous vaccination campaigns conducted in DRC and elsewhere, and rapidly declined over a 3-year period following a mass vaccination campaign in Uvira. While emigration of vaccinated individuals, immigration of unvaccinated individuals and births and deaths contributed to reducing vaccination coverage in Uvira over the research period, a substantial fraction of the population was not vaccinated in the campaign. Improving acceptance and uptake of kOCVs in future campaigns will require strategies that expand vaccine access times (e.g., weekends) and engage with influential community leaders to build confidence in vaccine safety.

Our estimates of initial campaign coverage are well below estimates of administrative coverage following the campaign and are lower than campaign vaccination coverage of kOCVs in other regions of the DRC^19^ and other countries^20 21^ prior to COVID-19. The coverage estimates are also lower than initial campaign vaccination coverage for kOCV campaigns conducted during the pandemic in Uganda^22^ and Cameroon.^23^ The vaccination campaign in Uvira happened only a month after the official end of the 2018–2020 and the largest Ebola outbreak recorded in DRC, which was associated with widespread institutional distrust and misinformation in the region.^24^ This, on top of rumours around COVID-19, is likely to have substantially contributed to lower vaccine acceptability in the population. The discrepancy between administrative coverage and our estimates may also reflect inaccuracies in the population counts (i.e., the denominator) for each health area that were used for vaccine planning.

The spatial variation in coverage could be due to the clustering of individuals with low vaccine confidence in specific neighbourhoods or due to different local vaccination team dynamics as vaccinators were recruited and trained separately, and how the campaign was organised. The top three reasons for non-vaccination included reasons associated with vaccine confidence (e.g., fear of side effects) as well as vaccine access (e.g., being away from home at the time of the campaign). During the survey, our study enumerators anecdotally reported collective refusals to vaccinate in certain avenues where vaccinators were chased away due to rumours about kOCVs. Opening vaccination sites and scheduling door-to-door visits earlier in the morning, during weekends or during public holidays may increase coverage among individuals who are away from home during the workday (e.g., workers, students, etc.). A higher proportion of individuals in higher wealth quintiles were very or moderately concerned that the vaccine would cause them to have a serious reaction compared with lower wealth quintiles (online supplemental material S9), potentially reflecting differences in exposures to mis/disinformation in internet or social media.^25^ These findings emphasise that education campaigns promoting vaccine confidence should not be targeted only based on socioeconomic status.^25^ A higher proportion of vaccinated individuals self-reported having seen or heard rumours about cholera vaccines, though this may reflect that unvaccinated individuals consume information that they are not aware is a rumour or that vaccinated individuals are more interested in health information and better remember hearing rumours. Vaccine confidence is context-specific and can vary over time;^26^ therefore, barriers and facilitators of vaccine uptake in Uvira may have limited the generalisability to other cholera-endemic settings. Rapid community assessments prior to vaccination campaigns can help identify interventions most likely to improve vaccine confidence in each unique setting.^27^

It is critical that activities to build vaccine confidence occur prior to additional vaccination campaigns in Uvira, given that 30% of unvaccinated individuals reported being unsure or little or not at all likely to accept kOCVs if an additional campaign was conducted. Based on the results of this analysis, the following strategies should be considered to build vaccine confidence in Uvira (and similar areas) prior to future vaccination campaigns: (1) working with influential community and religious leaders to disseminate messages in community meetings that build confidence in vaccine safety; (2) working with locally influential healthcare providers and news and radio stations to offer regular, live ‘office hours’ when viewers and listeners can have their questions answered and updated information shared; (3) identifying ‘champions’ who got vaccinated to share their stories on the news and in the workplace about why they got vaccinated and their experiences with side effects (e.g., “I felt a headache and tiredness on the day I was vaccinated, but the next day I felt back to my full health”) and (4) inviting community representatives to join a health area vaccine confidence task force to help develop appropriate community engagement strategies.^27^ Future qualitative research can help understand barriers to vaccine confidence in greater depth and further inform the design of targeted interventions.

Our findings have implications for the frequency of preventative campaigns needed to sustainably interrupt cholera transmission in cholera hotspots. Our estimates of coverage decline over the 3 years following the campaign are consistent with declines observed following a cluster-randomised controlled trial for kOCVs in Bangladesh where two-dose coverage declined from 66% at baseline to 48% after 1 year, 39% after 2 years, 31% after 3 years and 28% after 4 years.^28^ Applying our exponential decay model to these data, we estimate a mean yearly decay rate of kOCV coverage of 24% (95% credible interval: 19 to 27) following the trial in Bangladesh (compared with our estimated decay rate of 18% per year in Uvira). Similar to the findings in Bangladesh, migration out of the study area likely contributed to declines in coverage in Uvira over time.^28^ Migration of unvaccinated individuals into the study area, from unvaccinated areas in the region or from neighbouring countries such as Burundi, likely also contributed to declines in coverage. This could explain, at least partly, the difference observed between the kOCV coverage estimates from our surveys and estimates from surveys conducted earlier in Uvira. A rapid vaccination coverage monitoring survey conducted during the campaign suggested that 57.3% of the population received two doses of kOCV in the entire Uvira health zone, of which the city of Uvira is part. Another household survey conducted 4 months after vaccination reported a two-dose vaccination coverage of 56.6% for Uvira health zone (with no estimates provided for a single-dose coverage) (unpublished data collected by the National Program for the Elimination of Cholera and the Control of Other Diarrheal Disease), which is notably higher than our model-based estimates of two-dose coverage 4 months after the campaign (37%; 95% credible interval 29% to 45%). In addition to population movement, those discrepancies may reflect the differences in survey design and sampling approaches, with our estimates only covering the city, not the entire Uvira Health Zone. Given the important role of indirect protection from oral cholera vaccines, maintaining high coverage in the population may be key to sustained interruptions of transmission. Synchronous vaccination campaigns, sometimes across national borders (e.g., in Burundi in the case of Uvira), may be beneficial to prolonging these indirect effects, ideally leading to revaccination campaigns occurring on a similar timescale to the waning of direct protection (i.e., 5 years or more).

This study has several limitations. Our sampling framework was based on detecting structures built from recent satellite imagery. Using this approach, we were not able to sample households proportionate to their size, which, if correlated with vaccination coverage, could have led to bias in our estimates that could cause overestimation or underestimation of coverage. Our analyses accounted for household-level clustering and should therefore minimise the potential for such bias. Although we used visual aids to try to improve the specificity of questions related to the receipt of kOCVs, we assessed coverage several months/years after the campaign. The occurrence of other vaccination campaigns in Uvira (e.g., vaccines against COVID-19, measles, polio, etc.) and the lack of specificity in reporting kOCVs could lead to an overestimation of coverage. A small number of children in our surveys (n=51) had caregivers or survey respondents that reported the child was vaccinated, despite being age-ineligible (<1 year of age) at the time of the campaign. This may be due to issues with poor recall, social desirability, or due to ineligible children being vaccinated during the campaign though we are unable to distinguish between these with available data. In later survey rounds, there may have been more recall bias due to the longer time since vaccination, though the direction of this bias is unclear. Based on discussions with community health workers and nurses who participated in the 2020 vaccination campaign, parents in some households, particularly those who had experienced cholera in the past, were insistent that children below 1 year of age also receive the vaccine and, in some cases, vaccines were provided to this group. The use of vaccination registers in future campaigns could significantly improve the accuracy of coverage estimates and drivers of coverage decline over time.

While we examined differences in vaccine confidence by the number of kOCV doses received, our cross-sectional design makes it difficult to assess the temporal changes in attitudes towards the vaccine. For example, individuals may have refused the vaccine in the first round of the campaign due to fears about vaccine safety but later accepted their first dose during the second round of the campaign after seeing the vaccine’s safety in members of their community. Changes in vaccine access between campaign rounds may have also occurred because of varying vaccination dynamics across neighbourhoods. Longitudinal research that examines vaccination attitudes across multiple time points can help better tease apart reasons for incomplete vaccination.

In survey rounds 2 and 3, we only enrolled individuals in households where at least one person consented to a blood draw. The health seeking behaviour and willingness to receive the vaccine in households consenting to blood draw may be different from those where no one accepts to give a blood sample; living in households participating in serological surveys may have a different cholera risk perception and may be more willing to receive the vaccine than those in households refusing to participate in cholera serosurveys. That may lead to an overestimation of the vaccination coverage.

Our results suggest that while population movement partly contributes to the decay in vaccination coverage in Uvira over time, an important fraction of the population was not vaccinated in the 2020 campaigns. This highlights the challenges of conducting mass vaccination campaigns during the COVID-19 pandemic period and the impact of population movements diluting effective vaccination coverage following mass vaccination. Improving acceptance and uptake of kOCVs in future campaigns will require strategies that expand vaccine access times to span weekends and involve context-specific interventions that build confidence in vaccine safety. While difficult in practice due to limited vaccine availability, future campaigns should consider targeting larger spatially contiguous areas, and in the case of Uvira, including cross-border campaigns.

## Supplementary material

10.1136/bmjph-2024-001035 online supplemental file 1

10.1136/bmjph-2024-001035 online supplemental file 2

10.1136/bmjph-2024-001035 online supplemental file 3

10.1136/bmjph-2024-001035 online supplemental file 4

10.1136/bmjph-2024-001035 online supplemental file 5

10.1136/bmjph-2024-001035 online supplemental file 6

10.1136/bmjph-2024-001035 online supplemental file 7

10.1136/bmjph-2024-001035 online supplemental file 8

10.1136/bmjph-2024-001035 online supplemental file 9

## Data Availability

Data are available in a public, open-access repository.

## References

[1] World Health Organization (2017). Cholera vaccines: who position paper.

[2] Bi Q, Ferreras E, Pezzoli L (2017). Protection against cholera from killed whole-cell oral cholera vaccines: a systematic review and meta-analysis. Lancet Infect Dis.

[3] World Health Organization (2023). Weekly epidemiological record.

[4] Shortage of cholera vaccines leads to temporary suspension of two-dose strategy, as cases rise worldwide. https://www.who.int/news/item/19-10-2022-shortage-of-cholera-vaccines-leads-to-temporary-suspension-of-two-dose-strategy--as-cases-rise-worldwide.

[5] Bhattacharya SK, Sur D, Ali M (2013). 5 year efficacy of a bivalent killed whole-cell oral cholera vaccine in Kolkata, India: a cluster-randomised, double-blind, placebo-controlled trial. Lancet Infect Dis.

[6] Khatib AM, Ali M, von Seidlein L (2012). Effectiveness of an oral cholera vaccine in Zanzibar: findings from a mass vaccination campaign and observational cohort study. Lancet Infect Dis.

[7] Peak CM, Reilly AL, Azman AS (2018). Prolonging herd immunity to cholera via vaccination: Accounting for human mobility and waning vaccine effects. PLoS Negl Trop Dis.

[8] Cholera - Democratic Republic of the Congo. https://www.who.int/emergencies/disease-outbreak-news/item/2023-DON441.

[9] Lessler J, Moore SM, Luquero FJ (2018). Mapping the burden of cholera in sub-Saharan Africa and implications for control: an analysis of data across geographical scales. Lancet.

[10] Rebaudet S, Sudre B, Faucher B (2013). Environmental determinants of cholera outbreaks in inland Africa: a systematic review of main transmission foci and propagation routes. J Infect Dis.

[11] Plan Stratégique Multisectoriel d’Elimination du Choléra et contrôle des autres maladies diarrhéiques en République Démocratique du Congo 2023-2027 (Not yet published).

[12] Tiede D, Schwendemann G, Alobaidi A (2021). Mask R-CNN-based building extraction from VHR satellite data in operational humanitarian action: An example related to Covid-19 response in Khartoum, Sudan. Trans GIS.

[13] World Health Organization (2022). Behavioural and social drivers of vaccination: tools and practical guidance for achieving high uptake.

[14] Gabry J, Češnovar R, Bales B (2022). R Interface to CmdStan. https://mc-stan.org/cmdstanr/.

[15] Malembaka EB, Bugeme PM, Hutchins C (2024). Effectiveness of one dose of killed oral cholera vaccine in an endemic community in the democratic republic of the congo: a matched case-control study. Lancet Infect Dis.

[16] Filmer D, Pritchett LH (2001). Estimating wealth effects without expenditure data—or tears: An application to educational enrollments in states of India. Demography.

[17] Koyuncu A, Bugeme1 PM, Hulse1 JD (2024). Challenges with achieving and maintaining high oral cholera vaccine coverage in uvira, the democratic republic of the congo: serial cross-sectional representative surveys. Open Science Framework.

[18] United States Census Burea International database. https://www.census.gov/data-tools/demo/idb/#/pop?COUNTRY_YEAR=2023&COUNTRY_YR_ANIM=2023&CCODE_SINGLE=CD&CCODE=CD&popPages=PYRAMID&menu=popViz.

[19] Massing LA, Aboubakar S, Blake A (2018). Highly targeted cholera vaccination campaigns in urban setting are feasible: The experience in Kalemie, Democratic Republic of Congo. PLoS Negl Trop Dis.

[20] Baltazar CS, Rafael F, Langa JPM (2018). Oral cholera vaccine coverage during a preventive door-to-door mass vaccination campaign in Nampula, Mozambique. PLoS ONE.

[21] Grandesso F, Rafael F, Chipeta S (2018). Oral cholera vaccination in hard-to-reach communities, Lake Chilwa, Malawi. *Bull World Health Organ*.

[22] Bwire G, Kisakye A, Amulen E (2023). Cholera and COVID-19 pandemic prevention in multiple hotspot districts of Uganda: vaccine coverage, adverse events following immunization and WASH conditions survey. BMC Infect Dis.

[23] Amani A, Ngo Bama S, Dia M (2022). Challenges, best practices, and lessons learned from oral cholera mass vaccination campaign in urban Cameroon during the COVID-19 era. Vaccine (Auckl).

[24] Vinck P, Pham PN, Bindu KK (2019). Institutional trust and misinformation in the response to the 2018-19 Ebola outbreak in North Kivu, DR Congo: a population-based survey. Lancet Infect Dis.

[25] Hudson A, Montelpare WJ (2021). Predictors of Vaccine Hesitancy: Implications for COVID-19 Public Health Messaging. Int J Environ Res Public Health.

[26] MacDonald NE, SAGE Working Group on Vaccine Hesitancy (2015). Vaccine hesitancy: Definition, scope and determinants. Vaccine Auckl.

[27] Centers for Disease Control and Prevention (2021). COVID-19 vaccine confidence rapid community assessment guide.

[28] Ali M, Qadri F, Kim DR (2021). Effectiveness of a killed whole-cell oral cholera vaccine in Bangladesh: further follow-up of a cluster-randomised trial. Lancet Infect Dis.

[29] (2018). JMP methodology 2017 update & SDG baselines.

