# Supplementary material for: Challenges with achieving and maintaining oral cholera vaccine coverage: insights from serial cross-sectional representative surveys in a cholera-endemic community in the Democratic Republic of the Congo
Source: BMJ Public Health. 2025 Jan 19;3(1):e001035. doi: 10.1136/bmjph-2024-001035 (PMC11812865; doi:10.1136/bmjph-2024-001035)
Supplement: online supplemental file 2 [file bmjph-3-1-s002.pdf]

S2. Questions from the household survey related to population movement, Uvira, 2021-2023

Round 1	Round 2	Round 3	Question
X	X	X	How many individuals are part of this household?
X	X*	X*	How many individuals were part of your household in the last year who are no longer staying here (but are still alive)?*
X	X	X**	(Apart from births), how many current household members were not members of your household one year ago?**
	X	X	Of the individuals who were part of your household in the last year who are no longer staying here, how many have left Uvira town?
	X	X	Of the current household members who were not members of your household one year ago, how many came from outside Uvira?

*The question was modified to end with “but are still alive”.
**The question was modified to begin with “Apart from births”.
