# Supplementary material for: Challenges with achieving and maintaining oral cholera vaccine coverage: insights from serial cross-sectional representative surveys in a cholera-endemic community in the Democratic Republic of the Congo
Source: BMJ Public Health. 2025 Jan 19;3(1):e001035. doi: 10.1136/bmjph-2024-001035 (PMC11812865; doi:10.1136/bmjph-2024-001035)
Supplement: online supplemental file 3 [file bmjph-3-1-s003.pdf]

S3. Perceptions about cholera and cholera vaccines between partially vaccinated (1 dose) and fully vaccinated (2 or more doses) adults, Uvira, 2022

Perceptions		Partially Vaccinated: 1-dose	Fully Vaccinated: ≥ 2 doses	χ^2 p-value
I will probably get sick with cholera				
	Strongly agree	86% (258)	87% (203)	
	Somewhat agree	11% (33)	12% (28)	0.22
	Neither agree nor disagree	3% (9)	1% (2)	
	Don't agree	0% (0)	0% (0)	
Getting sick with cholera can be serious				
	Strongly agree	90% (332)	91% (249)	
	Somewhat agree	9% (32)	8% (23)	0.93
	Neither agree nor disagree	1% (3)	1% (3)	
	Don't agree	0% (0)	0% (0)	
How important is a cholera vaccine to protect you against cholera				
	Very important	90% (346)	93% (269)	
	Moderately important	8% (32)	6% (18)	0.36
	Little important	1% (5)	1% (2)	
	Not important	1% (2)	0% (0)	
How safe do you think a cholera vaccine is for you				
	Very safe	74% (262)	75% (194)	
	Moderately safe	20% (71)	22% (57)	0.19
	Little safe	6% (22)	3% (8)	
	Not at all safe	0% (0)	0% (0)	

How concerned are you that a cholera vaccine could cause you to have a serious* reaction				
	Not concerned	64% (239)	66% (185)	0.26
	Little concerned	9% (34)	8% (21)	
	Moderately concerned	18% (67)	14% (38)	
	Very concerned	10% (36)	13% (36)	
How much do you trust the public health agencies that recommend the cholera vaccine[A]				
	Fully trust	72% (134)	76% (112)	0.48
	Mostly trust	22% (42)	19% (28)	
	Somewhat trust	6% (11)	4% (6)	
	Do not trust	0% (0)	1% (1)	
Perceptions about how many family members are vaccinated**				
	Almost all	20% (72)	32% (88)	0.002
	Many	41% (147)	39% (108)	
	Somewhat agree	39% (139)	28% (77)	
	None	1% (3)	2% (5)	
Perceptions about how many community and religious leaders are vaccinated***				
	Almost all	8% (18)	15% (29)	0.04
	Many	28% (65)	32% (61)	
	Somewhat agree	51% (116)	45% (85)	
	None	13% (30)	8% (16)	

Data are proportion (n).

*Serious means you would not be able to perform your daily activities

[A] Only asked to subset of participants who were familiar with public health agencies recommending vaccines (N=187 partially vaccinated and N=147 fully vaccinated). ** The question was: “If you had to guess, about how many of your

family and friends have received a cholera vaccine?”. ***The question was: “If you had to guess, about how many of your community leaders or religious leaders have received a cholera vaccine?”.
