# Supplementary material for: Challenges with achieving and maintaining oral cholera vaccine coverage: insights from serial cross-sectional representative surveys in a cholera-endemic community in the Democratic Republic of the Congo
Source: BMJ Public Health. 2025 Jan 19;3(1):e001035. doi: 10.1136/bmjph-2024-001035 (PMC11812865; doi:10.1136/bmjph-2024-001035)
Supplement: online supplemental file 4 [file bmjph-3-1-s004.pdf]

S4. Self-reported reasons for non-vaccination among individuals who received zero doses of killed oral cholera vaccines, Uvira, 2022

Reason	%* (N)
I suspected the vaccine was containing the COVID-19, Ebola, or other microbes	35% (675)
I feared the side effects	18% (356)
I was away from home when vaccinators passed by	26% (492)
I was not eligible for the vaccine	12% (224)
Vaccinators did not arrive in my avenue	11% (220)
I thought the vaccine was inefficacious	8% (159)
Lack of trust in health system	7% (128)
Other	6% (106)
Vaccinators arrived in my avenue but not at my home	3% (66)
Fertility concerns	3% (65)
My spouse/the decision maker is not in favor of vaccination	2% (44)
Don’t know	2% (30)
Religious concerns	0.3% (5)

*Percentages will not add to 100% because the survey question was select all that apply
