# Supplementary material for: Challenges with achieving and maintaining oral cholera vaccine coverage: insights from serial cross-sectional representative surveys in a cholera-endemic community in the Democratic Republic of the Congo
Source: BMJ Public Health. 2025 Jan 19;3(1):e001035. doi: 10.1136/bmjph-2024-001035 (PMC11812865; doi:10.1136/bmjph-2024-001035)
Supplement: online supplemental file 5 [file bmjph-3-1-s005.pdf]

S5. Killed oral cholera vaccine (kOCV) coverage in each survey, Uvira, 2021-2023

Round	N	Months after 2nd dose campaign*	One dose (95% CI)	Two doses (95% CI)
Survey 1	2292	10.1 - 10.7	32% (38-36)	23% (20-27)
Survey 2	3581	17.9 – 18.9	27% (24-29)	20% (17-23)
Survey 3	2768	29.8 – 30.5	28% (25-31)	10% (8-12)

Excludes 98 individuals who were vaccinated but did not know the number of doses.

*The second dose campaign was completed on October 2, 2020
