# Supplementary material for: Challenges with achieving and maintaining oral cholera vaccine coverage: insights from serial cross-sectional representative surveys in a cholera-endemic community in the Democratic Republic of the Congo
Source: BMJ Public Health. 2025 Jan 19;3(1):e001035. doi: 10.1136/bmjph-2024-001035 (PMC11812865; doi:10.1136/bmjph-2024-001035)
Supplement: online supplemental file 6 [file bmjph-3-1-s006.pdf]

S6. Population-weighted mean coverage of at least one dose of killed oral cholera vaccine (kOCV) in each survey, Uvira, 2021-2023

	Round 1	Round 2	Round 3
Kabindula	53.4 (36.0, 70.7)	26.4 (17.2, 35.5)	34.0 (20.2, 47.9)
Kakombe	73.6 (61.6, 85.6)	60.0 (51.5, 68.4)	50.0 (42.9, 57.1)
Kalundu	60.8 (47.8, 73.8)	30.9 (22.6, 39.3)	22.0 (15.0, 29.0)
Kasenga	56.1 (44.9, 67.4)	55.3 (46.7, 63.8)	43.2 (33.3, 53.1)
Kavimvira	67.2 (57.8, 76.5)	61.0 (53.8, 68.3)	42.5 (33.4, 51.5)
Kibondwe	78.3 (49.8, 106.7)	48.8 (29.9, 67.6)	62.0 (46.6, 77.5)
Kilibula	53.2 (31.6, 74.9)	26.4 (15.7, 37.1)	18.6 (7.7, 29.6)
Kimanga	45.9 (26.0, 65.8)	47.1 (24.1, 70.0)	25.6 (7.9, 43.4)
Mulongwe	39.5 (24.6, 54.4)	28.1 (14.8, 41.5)	47.1 (29.8, 64.4)
Nyamianda	23.7 (10.7, 36.6)	21.1 (8.7, 33.5)	26.4 (11.1, 41.7)
Rombel I	29.4 (-4.8, 63.6)	47.0 (29.7, 64.3)	57.9 (32.3, 83.5)
Rombe II	46.7 (31.8, 61.7)	49.3 (38.2, 60.5)	40.1 (26.4, 53.9)
Rugenge	59.4 (41.8, 76.9)	58.6 (48.8, 68.5)	50.0 (27.0, 73.0)
Songo	55.8 (38.7, 72.8)	42.2 (26.6, 57.7)	42.3 (27.6, 57.0)

Data are mean coverage estimate (95% confidence interval).
