# Supplementary material for: Challenges with achieving and maintaining oral cholera vaccine coverage: insights from serial cross-sectional representative surveys in a cholera-endemic community in the Democratic Republic of the Congo
Source: BMJ Public Health. 2025 Jan 19;3(1):e001035. doi: 10.1136/bmjph-2024-001035 (PMC11812865; doi:10.1136/bmjph-2024-001035)

S7. Smoothed estimates of at least one dose of killed oral cholera vaccine coverage (%) across Uvira by survey round with sampled points shown in black and boundaries of health areas in grey, Uvira, 2021-2023

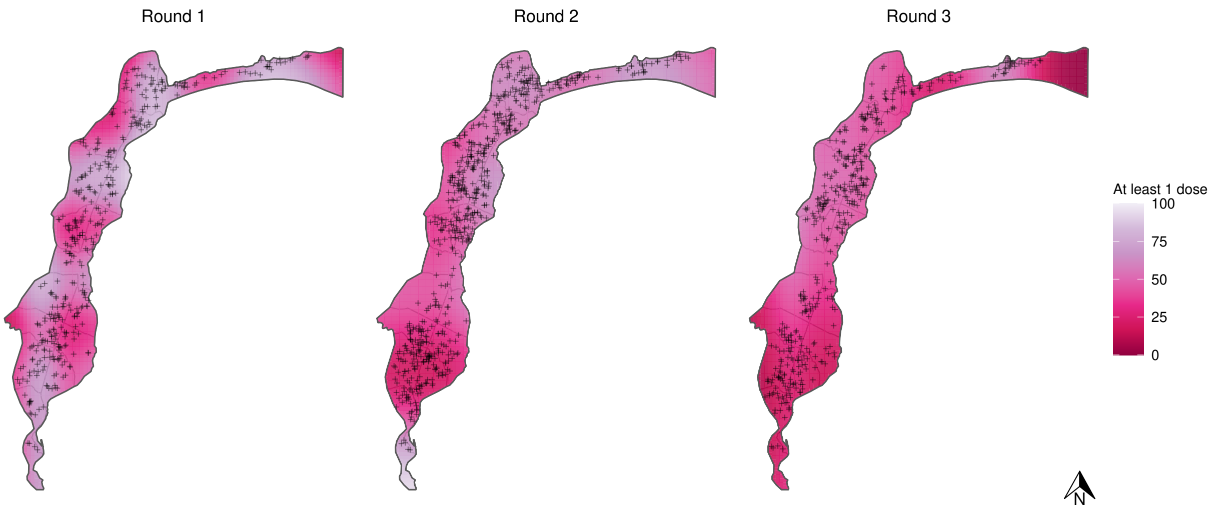

Supplement: online supplemental file 7 [file bmjph-3-1-s007.pdf]
