# Supplementary material for: Challenges with achieving and maintaining oral cholera vaccine coverage: insights from serial cross-sectional representative surveys in a cholera-endemic community in the Democratic Republic of the Congo
Source: BMJ Public Health. 2025 Jan 19;3(1):e001035. doi: 10.1136/bmjph-2024-001035 (PMC11812865; doi:10.1136/bmjph-2024-001035)

S8. Projected coverage of at least two doses of killed oral cholera vaccine over time for the overall population and by age, Uvira, 2021-2023. Points indicate overall and age-stratified survey coverage estimates. Dashed lines indicate survey rounds.

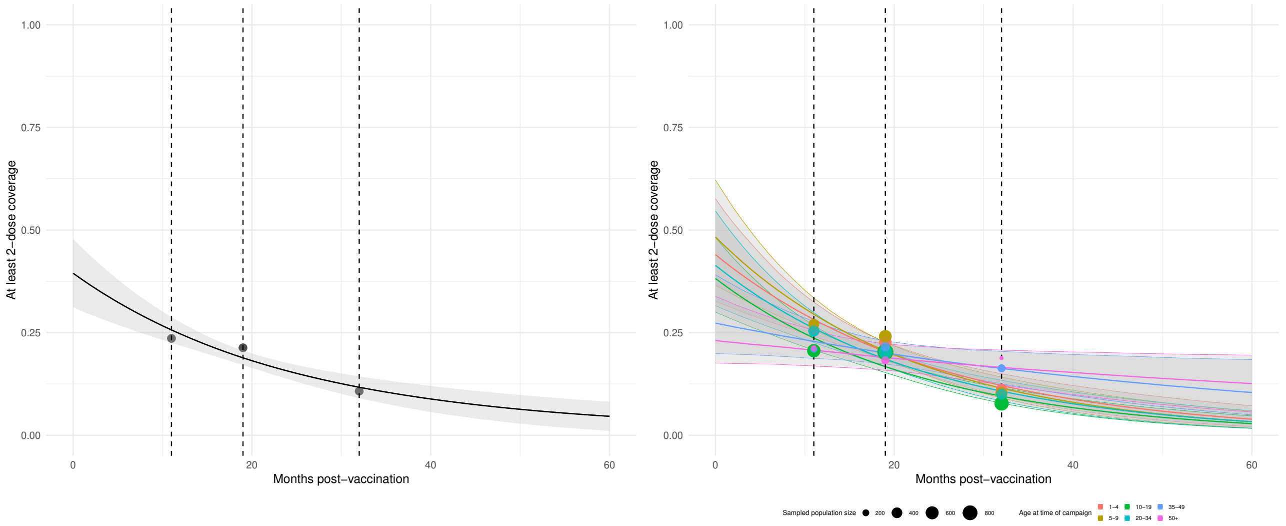

Supplement: online supplemental file 8 [file bmjph-3-1-s008.pdf]
