# Supplementary material for: Challenges with achieving and maintaining oral cholera vaccine coverage: insights from serial cross-sectional representative surveys in a cholera-endemic community in the Democratic Republic of the Congo
Source: BMJ Public Health. 2025 Jan 19;3(1):e001035. doi: 10.1136/bmjph-2024-001035 (PMC11812865; doi:10.1136/bmjph-2024-001035)
Supplement: online supplemental file 9 [file bmjph-3-1-s009.pdf]

S9. Perceptions about cholera and cholera vaccines by wealth quintile, Uvira, 2022

	Wealth Quintile				
Perceptions	**1st (lowest)**	**2nd**	**3rd**	**4th**	**5th (highest)**
I will probably get sick with cholera					
Strongly agree	84%	84%	87%	74%	96%
Somewhat agree	12%	13%	12%	21%	2%
Neither agree nor disagree	4%	3%	1%	4%	2%
Don't agree	0%	0%	0%	0%	0%
Getting sick with cholera can be serious					
Strongly agree	85%	84%	88%	80%	88%
Somewhat agree	12%	13%	11%	18%	11%
Neither agree nor disagree	3%	3%	1%	2%	1%
Don't agree	0%	0%	0%	0%	0%
How important is a cholera vaccine to protect you against cholera					
Very important	83%	80%	80%	73%	75%
Moderately important	8%	13%	13%	21%	18%
Little important	5%	3%	4$	4%	4%
Not important	4%	5%	3%	2%	3%
How safe do you think a cholera vaccine is for you					
Very safe	69%	61%	64%	64%	66%
Moderately safe	19%	30%	28%	29%	27%
Little safe	12%	9%	8%	7%	7%
Not at all safe	0%	0%	0%	0%	0%
How concerned are you that a cholera vaccine could cause you to have a serious* reaction					

Not concerned	57%	53%	50%	45%	37%
Little concerned	13%	10%	10%	12%	16%
Moderately concerned	17%	21%	21%	23%	21%
Very concerned	13%	15%	18%	20%	25%
How much do you trust the public health agencies that recommend the cholera vaccine[A]					
Fully trust	66%	60%	65%	62%	62%
Mostly trust	18%	31%	25%	26%	32%
Somewhat trust	9%	5%	8%	12%	4%
Do not trust	7%	4%	1%	1%	3%
Perceptions about how many family members are vaccinated					
Almost all	13%	16%	15%	19%	5%
Many	31%	25%	28%	29%	24%
Somewhat agree	36%	40%	41%	39%	47%
None	21%	19%	15%	14%	23%
Perceptions about how many community and religious leaders are vaccinated					
Almost all	7%	6%	8%	11%	4%
Many	24%	20%	23%	16%	11%
Somewhat agree	46%	53%	51%	56%	60%
None	23%	21%	18%	18%	25%

*Serious means you would not be able to perform your daily activities

[A] Only asked to subset of participants who were familiar with public health agencies recommending vaccines (N=373 unvaccinated and N=334 vaccinated)
